# Paraquat poisoning: a case series of 15 survivors and narrative review

**DOI:** 10.1097/MS9.0000000000003174

**Published:** 2025-03-18

**Authors:** Sathish Sreenivas Pasam, Sameer Kumar Majety, Omar Nayeem, Devrakshita Mishra, Sandeep Chakra G, Riya Singh, Mallikarjuna Praneeth Karuchola, Aakash Anumolu

**Affiliations:** a Department of General Medicine, Rangaraya Medical College, Kakinada, India; b School of Medicine, Xiamen University, Xiamen, PR China; c School of Medicine, University of Perpetual Help System Delta, Laspinas, Philippines; d Bukovinian State Medical University, Chernvtsi, Ukraine

**Keywords:** neurotoxicity of paraquat, paraquat and hemodialysis, paraquat and n-acetyl cysteine, paraquat poisoning, paraquat prognosis

## Abstract

**Background:**

Paraquat (PQ) poisoning is a grave concern in developing countries due to its wide availability. Acute paraquat poisoning can have both systemic and local manifestations, with mortality rates that can reach as high as 90%; pulmonary complications and multiple organ dysfunction syndromes being major causes. This case series is a unique retrospective observational study of 15 survivors from South India.

**Case presentation:**

The case series consists of 15 cases, with a mean age of 24.6 years (excluding outliers), that were alleged to have taken varying amounts of paraquat dichloride. Patients exhibited a diverse range of symptoms affecting multiple organ systems, with particular emphasis on kidney, liver, and lung function. Treatments included a combination of hemodialysis, targeted drug therapy in the form of N-acetyl cysteine, anti-inflammatory therapy with corticosteriods and symptomatic therapy. The case descriptions also include the details of the amount of paraquat allegedly ingested, the ingestion to hospitalization time, demographics, etc, that further help in determination of prognosis.

**Overview:**

PQ can cause a variety of clinical signs and symptoms, including gastrointestinal, renal, hepatic, and pulmonary problems. Less commonly, it can also affect the neurological and cardiac systems. Treatment is mainly focused on reducing the effective PQ concentration in blood, as no antidote has been named till date. The paper also discusses the various treatments available, drugs and procedures, and their mechanisms. Also prognostic factors like age, amount, ingestion to hospitalization time, etc.

**Conclusion:**

The study underlines the need for defined treatment protocols, prognostic factors, and enforcing restrictions on availability of this deadly poison.

## Background

Paraquat is a bipyridylium herbicide with the molecular formula C12H14Cl2N2 (1,1ʹ-dimethyl-4,4ʹ-bipyridylium dichloride). Commercially, sold as gramoxone, this herbicide is widely used in a variety of crops such as cotton, citrus, etc^[1]^. It has extremely toxic and detrimental effects on multiple organ systems including the renal, pulmonary, hepatobiliary, and gastrointestinal systems, the former two being fatal. In the Indian subcontinent, self-consumption of paraquat is a leading cause of morbidity and mortality. The most common cause of mortality is due to pulmonary complications or multiple organ dysfunction syndrome or multi-organ dysfunction syndrome (MODS). After ingestion, it gets absorbed through the GI tract, skin, and respiratory tract^[2]^.

**HIGHLIGHTS**

- Paraquat poisoning is a growing concern due to its remarkably high mortality in many countries that still have not restricted/banned its usage. This paper is a retrospective observational study of 15 unique such cases of acute paraquat poisoning that survived, with the help of intensive care and treatment.
- It discusses the various subjects within paraquat poisoning like the numerous clinical findings, current treatment modalities, possible prognostic factors, mortality, etc, with respect to the cases in the form of a narrative review.
- Some of the significant topics discussed include the mechanism of action of paraquat, common clinical findings like the features of pulmonary, renal, and hepatic complications, the role of hemodialysis and drugs like N-acetyl cysteine, dexamethasone, cyclophosphamide, etc, within the current treatment modalities, etc.
- Few possible prognostic factors like shorter ingestion to hospitalization time, early initiation of immunosuppressive therapy and dialysis might have played a role in their good prognosis.
- This paper highlights the need for defined treatment protocols and prognostic factors in the management of paraquat cases. It also brings to the forefront the role of a multidisciplinary team in such cases.

Paraquat undergoes metabolic transformation by various enzyme systems, including xanthine oxidase, NADPH-cytochrome reductase, nitric oxide synthase, and NADH-ubiquinone oxidoreductase. This process culminates in the formation of the paraquat mono-cation radical (PQ+) within cells. PQ+ is rapidly re-oxidized to PQ2+ and produces free radicals like superoxide (O2–), hydroxyl ions, and peroxynitrite (ONOO–) in the process and ultimately leads to fibrosis of the lungs^[3]^. It targets the lungs by affecting the polyamine transport system majorly expressed in the membrane of alveolar cells type 1, 2, and Clara cells. Pulmonary concentrations of paraquat are almost 6–10 folds higher than those in plasma. Successful treatments are available for initial stages but there is no effective way yet to stop its progression to pulmonary fibrosis^[4]^.

## Methods

Methods section for a case series of 15 paraquat poisoning survivors.

The work has been reported in line with the PROCESS criteria.

Study design: This was a retrospective case series study of patients admitted to a tertiary care hospital in southern India.

Inclusion criteria:

- Patients diagnosed with paraquat poisoning based on clinical presentation, laboratory findings, and/or history of paraquat ingestion.

Exclusion criteria:

- Patients with incomplete medical records.
- Patients with concurrent poisoning or severe underlying medical conditions that could confound the assessment of paraquat poisoning.

Data collection:

- Patient demographic data, including age, sex, and occupation.
- History of paraquat ingestion, including dose, route, and time elapsed.
- Clinical presentation at admission, including symptoms, signs, and vital signs.
- Laboratory findings, including complete blood count, renal function tests, and liver function tests.
- Treatment modalities, including supportive care, dialysis, and any specific antidotes or therapies.
- Outcome data, including length of hospital stay, complications, and long-term follow-up.

Data analysis:

- Descriptive statistics were used to summarize patient characteristics, clinical presentation, and outcomes.
- Frequencies and percentages were calculated for categorical variables.
- Patient confidentiality was maintained throughout the study.
- Informed consent was obtained from patients or their legal representatives, when appropriate.
- Institutional review board approval was not required for case series in our hospital.

This case series has been documented in accordance with the PROCESS guidelines for reporting case series^[5]^.

## Case presentation

### Case 1

A 43-year-old female patient was brought to the hospital for suspected self-consumption of an unknown amount of paraquat dichloride taken around 6 hours before admission. The vitals were stable on presentation, however the patient had two episodes of vomiting. The patient also complained of severe throat pain and odynophagia 1 day after admission. The patient had both renal and pulmonary complications associated with paraquat poisoning; renal complications in the form of an acute kidney injury (AKI) with the serum creatinine peaking at 5.4 mg/dL. She received four rounds of dialysis for the same, with the final post-dialysis serum creatinine level at 1.7 mg/dL. For the pulmonary complications, the patient underwent a chest computed tomography (CT), the findings of which were subpleural reticular densities in right upper, middle, bilateral lower lobes and left lower lingula along with mild pleural thickening on the right side indicating that the patient was in the initial stages of pulmonary fibrosis. The administered treatment, including symptomatic and targeted therapy, is presented in Table 5. As the patient had already received treatment for the above-mentioned complications and no new incipient complications were observed, she was discharged after 21 days of treatment under stable conditions.

**Table 5:** Case-specific pharmacological treatment of paraquat poisoning; symptomatic treatment includes – stomach wash, PPI, prophylactic antibiotics to avoid secondary infections, and IV fluids

Case	N–acetyl cysteine	Methylprednisolone	Dexamethasone	Cyclophosphamide	Symptomatic treatment and Vit A, C, and E
1	+	+		+	+
2	+	+			+
3	+		+	+	+
4	+	+	+	+	+
5	+	+			+
6	+	+		+	+
7	+	+	+		+
8	+	+		+	+
9	+	+	+		+
10		+	+	+	+
11	+	+		+	+
12	+	+	+		+
13	+	+			+
14		+	+		+
15	+	+			+

### Case 2

A 27-year-old male patient was brought to the hospital for alleged consumption of an unknown quantity of paraquat poison mixed with soft drink. The patient presented to the local hospital with multiple episodes of non-bilious non-bloody vomiting with dysphagia, dry cough, left sided chest pain associated with fever and chills. Subsequently, he was referred to the current hospital 4 days after the initial paraquat consumption for AKI with serum creatinine peaking at 5.4 mg/dL. On examination, a whitish coat over the tongue was observed. Vitals were found to be stable. Dialysis procedures were done on the patient twice. The patient also experienced pulmonary complications associated with paraquat. A plain CT initially showed subpleural reticular densities in periphery of bilateral lung parenchyma and bilateral pleural thickening, which progressed into mild bilateral pleural effusion with subsegmental collapse over the course of the next 10 days. The patient was also referred to the Ophthalmology department for a complaint of continuous watery discharge from the eyes. The administered treatment, including symptomatic and targeted therapy, is presented in Table 5. As the patient was already treated for the above-mentioned complications and no new incipient complications were observed, he was discharged after 29 days of treatment under stable conditions.

### Case 3

A 25-year-old male was brought to casualty for suspected self-consumption of approximately 100 mL of paraquat dichloride 24% mixed with beer 19 hours before admission. The patient’s vitals were found to be stable on examination. The patient had 2–3 episodes of vomiting and loose stool immediately after ingestion. The patient later had epigastric and throat pain, green-colored urine and stool, and cough with expectoration. The patient’s urea and creatinine levels were slightly on the higher side upon admission with a urea level of 65 mg/dL and creatinine of 1.9 mg/dL. The patient underwent three sessions of dialysis, after which the urea and creatinine levels came to normal range. Liver function tests (LFTs) were normal with an average total bilirubin (TB) of 0.7 mg/dL. The patient was given a chest CT for cough and throat pain and was found to have moderate to gross left pneumothorax with atelectasis of the lower left lobe and small atelectatic bands in the upper right lobe. He was then referred to the pulmonology department. The administered treatment, including symptomatic and targeted therapy, is presented in Table 5. Due to the absence of any new emerging complications along with the successful resolution of previous complications, he was discharged following a 20-day treatment period under stable conditions.

### Case 4

A 26-year-old male was brought to the ER for suspected self-consumption of approximately 10 mL of paraquat taken 6 hours before admission. The vitals were stable on presentation. The patient complained of one episode of blood-stained vomiting and being unable to eat or drink. On physical examination, the patient had a dry oral cavity with hoarseness of voice and epigastric tenderness. The patient experienced renal complications associated with paraquat poisoning in the form of an AKI having an initial serum creatinine level of 2.2 mg/dL, for which the patient was given four rounds of hemodialysis. Each of which had a post-dialysis serum creatinine level of 4.8, 4.0, and 3.0 mg/dL, respectively. The patient also experienced an acute liver injury, with the TB peaking at 1.4 mg/dL and reducing to 0.4 mg/dL with supportive liver treatment. The patient had no pulmonary complications. The administered treatment, including symptomatic and targeted therapy, is presented in Table 5. As the patient had been treated successfully for the previous complications and no incipient complications were observed and the patient appeared to be stable, he was discharged after 8 days of treatment.

### Case 5

A 22-year-old male patient was brought to the hospital for suspected self-consumption of approximately 20 mL of paraquat dichloride taken 75 hours (about 3 days) before admission. The patient’s vitals were found to be stable on examination. The patient had three episodes of vomiting, odynophagia, and oral ulcers; and later developed throat pain, abdominal discomfort, and dry cough. The patient was associated with AKI and LRTI with the presence of tubular breath sounds. The patient’s urea level measured 118 mg/dL and creatinine 5.9 mg/dL upon admission. Subsequently, the patient underwent dialysis for 3 hours on the same day, followed by another session 4 days later. After both dialysis sessions, the urea and creatinine levels returned to within the normal range. The patient’s TB was well within the normal range with an average TB of 0.7 mg/dL. The patient was referred to the pulmonology department due to a lower respiratory tract infection. The administered treatment, including symptomatic and targeted therapy, is presented in Table 5. He was discharged following a 14-day treatment period, having recovered from AKI and maintaining stable conditions.

### Case 6

A 33-year-old male was brought to casualty for suspected consumption of approx. Thirty milliliters of paraquat 3 hours before admission. The patient’s vitals were found to be stable on examination. The patient had two episodes of spontaneous vomiting and two to three episodes of induced vomiting along with loose stools. The patient was noted to have ulceration on local examination of the oral cavity. Upon admission, the patient exhibited elevated levels of urea and creatinine, with urea at 86 mg/dL and creatinine at 3.7 mg/dL. Following three sessions of hemodialysis, renal function tests (RFTs) returned to within the normal range. LFTs indicated normal results, with a TB level averaging 0.7 mg/dL. The patient’s cholinesterase level was high with a value of 13 778 U/L. The administered treatment, including symptomatic and targeted therapy, is presented in Table 5. After a few days of treatment, the patient also developed cough and constipation. Since no developing complications were detected, and the patient remained stable, he was discharged after undergoing 19 days of treatment.

### Case 7

A 19-year-old male patient was brought to the hospital with suspected self-ingestion of approximately 50 mL of paraquat taken 24 hours preadmission. The patient’s vitals showed a stable presentation. The patient complained of vomiting, pain in the oral cavity, and abdominal pain. On physical examination, the patient had mucosal erosions in the oral cavity with dysphagia and epigastric tenderness. The patient experienced renal complications associated with paraquat poisoning in the form of an AKI with the serum creatinine peaking at 2 mg/dL. The patient received four rounds of hemodialysis for the same, with the last post-dialysis serum creatinine level at 0.8 mg/dL. He was also found to have an acute liver injury, with a TB level of 1.4 mg/dL, and post-liver support treatment TB levels at 0.8 mg/dL. The patient had no pulmonary complications. The administered treatment, including symptomatic and targeted therapy, is presented in Table 5. As the patient was already treated for the above-mentioned complications and no new incipient complications were observed, he was discharged after 45 days of treatment under stable conditions.

### Case 8

This case is of a 24-year-old male patient who was brought to the hospital for suspected self-consumption of approximately 50 mL of paraquat dichloride taken 3 hours before admission. The patient’s vitals were found to be stable on examination. On physical examination, patient was found to be conscious, coherent, and without significant distress. The patient had two episodes of non-bloody vomiting. Although none of the pulmonary complications associated with paraquat poisoning were observed in the patient, the patient has been affected with acute liver injury, with a TB of 1.5 mg/dL, 2.5 mg/dL on two separate occasions and the patient recovered after receiving supportive treatment for the same. Although RFTs were normal during his initial period of the hospital stay, the 24-year-old experienced acute kidney injury with a serum creatinine level of 7.4 consequently three hemodialysis sessions were needed for this patient. The administered treatment, including symptomatic and targeted therapy, is presented in Table 5. As no incipient complications were observed and the patient was in stable condition, he was discharged after 15 days of treatment.

### Case 9

A 34-year-old male was brought to the hospital for alleged consumption of 10 mL of paraquat dichloride 4 hours before admission. The patient’s vitals were found to be stable on examination. The patient was having a burning sensation in his throat. Despite normal kidney function tests (RFTs) and LFTs upon admission, with urea at 28 mg/dL and creatinine at 0.9 mg/dL, TB was at 0.6 mg/dL. Prophylactically, two sessions of hemodialysis were performed, each lasting for 3 hours. The administered treatment, including symptomatic and targeted therapy, is presented in Table 5. The patient’s treatment course lasted 8 days, and he was discharged without any observed complications.

### Case 10

A 58-year-old male patient was brought to the hospital for suspected self-consumption of approximately 50 mL of paraquat dichloride taken 3 hours before admission. The patient’s vitals, and physical examination were found to be unremarkable. This patient did not experience any renal, hepatic, or pulmonary complications. All RFTs were normal during his hospital stay, with an average serum creatinine of 0.99 mg/dL, showing no evidence of renal injury. No hemodialytic session was needed for this patient. The administered treatment, including symptomatic and targeted therapy, is presented in Table 5. As no incipient complications were observed and the patient seeming to be stable, he was discharged after 7 days of treatment.

### Case 11

The following case is about a 17-year-old male patient who was brought to the hospital for suspected self-consumption of approximately 15 mL of paraquat dichloride taken 4 hours before admission. The patient’s vitals, and physical examination were found to be unremarkable. The patient had one episode of blood-stained vomiting. The patient did not experience any renal, pulmonary, or hepatic complications. All RFTs were normal during his hospital stay, with an average serum creatinine of 0.60 mg/dL, showing no evidence of renal injury. The patient was also referred to the psychiatry department due to his previous history of suicide attempts. The administered treatment, including symptomatic and targeted therapy, is presented in Table 5. No hemodialysis was needed for this patient. As no incipient complications were observed and the patient being stable, he was discharged after 3 days of treatment.

### Case 12

A 25-year-old female patient was brought to the hospital for suspected self-consumption of approximately 20 mL of paraquat dichloride taken 33 hours before admission to the local hospital. The patient was taken to this hospital after five episodes of self-induced vomiting, where she further had three more episodes of vomiting post a stomach wash. The patient’s physical examination was found to be unremarkable. The patient was then referred to our hospital for an AKI with serum creatinine level at 1.8 mg/dL, which further worsened, peaking at 2.9 mg/dL. Consequently, the patient underwent four rounds of dialysis. On examination, glossitis, oral mucosa congestion, and slough formation over the oral cavity and tongue were observed, Hence, the patient was sought an ENT Consultation. The administered treatment, including symptomatic and targeted therapy, is presented in Table 5. As no incipient complications were observed and the patient was under stable conditions, he was discharged after 18 days of treatment.

### Case 13

A 28-year-old male patient was brought to the hospital for suspected self-consumption of unknown amount of paraquat dichloride taken 12 hours before admission. The patient’s vitals were found to be stable on examination. On physical examination, the patient was found to be drowsy, gasping, having cold extremities, and left pupil dilated. The patient had one episode of non-bilious, non-bloody vomiting. Although none of the renal or pulmonary complications associated with paraquat poisoning were observed in the patient, 2 days after admission, the patient was found to have an acute liver injury, with a TB of 1.5 mg/dL. The patient recovered after receiving supportive treatment for the same. All RFTs were normal during his hospital stay, with an average serum creatinine of 0.76 mg/dL, showing no evidence of renal injury. A psychiatry consult was recommended due to his previous history of para-suicidal tendencies. The administered treatment, including symptomatic and targeted therapy, is presented in Table 5. As no incipient complications were observed and the patient was under stable conditions, he was discharged after 8 days of treatment.

### Case 14

A 38-year-old male was brought to the hospital under suspicion of self-ingestion of an unknown amount of paraquat dichloride taken 4 hours before admission. The vitals were stable on presentation. On physical examination, no specific or relevant findings were noted, however, on the third day of admission, the patient had a complaint of diffuse chest pain that came intermittently. The patient had none of the renal or pulmonary complications associated with paraquat. All values of renal and LFTs were normal throughout his treatment, with averages of TB at 0.6 mg/dL and serum creatinine levels at 0.7 mg/dL. No hemodialysis was needed for this patient. The administered treatment, including symptomatic and targeted therapy, is presented in Table 5. The patient was discharged after 6 days of therapy under stable conditions.

### Case 15

A 25-year-old male was brought to a local hospital for alleged self-consumption of approximately 30 mL of paraquat dichloride taken 4 hours before admission. The patient complained of throat pain and abdominal distress. The patient was noted to have oral ulceration over his tongue on physical examination, for which he was given mucaine gel. The patient was treated with a stomach wash and placed under observation for the day. The patient developed an AKI (serum Cr = 5.5 mg/dL) on his second day of admission. For which he was given one round of emergency dialysis. Once his creatinine had stabilized (1.7 mg/dL), he was transferred to our hospital for further management and specialist care, 7 days after the first consumption. Here he developed an acute liver injury (ALI) on the same day of admission, (TB = 2.3 mg/dL) and was given liver support for the same. On the second day, an HRCT was done, sub-pleural reticular densities in bilateral lower lobes and emphysematous changes were observed. On the third day, his serum creatinine levels rose again to 2.6 mg/dL, he received one round of hemodialysis for the same. By the fourth day, his serum TB and creatinine had stabilized (TB = 0.7 mg/dL, Cr = 0.9 mg/dL). The administered treatment, including symptomatic and targeted therapy, is presented in Table 5. As no incipient complications were observed and the patient was under stable conditions, he was discharged after 8 days of treatment in this hospital.

## Case discussion

### Mortality

Paraquat dichloride or C12H14N2+2 is a bipyridylium compound which is widely used as herbicide due to its efficacy and rapid onset action. A study conducted in North-Eastern India, with a sample size of more than 450 patients, highlighted the deadly nature of this herbicide; with a mortality rate of 94.74% among its consumers, However overall mortality in the study which included other means of suicide was 12%^[5]^. Another study based at a Tertiary care center in Southern India indicated that the in-hospital mortality rate was 72.7%^[6]^. Both the studies that were mentioned above are from different corners of the country, but the detrimental effects of the paraquat were evident in them due to the extensive availability to this poison throughout India. A retrospective study focused on herbicidal poisonings conducted in a tertiary care center stated that paraquat contributed to 78.3% of overall herbicidal ingestions and also that students were the most affected demographic followed by farmers^[7]^. These examples highlight the accessibility of this compound to an atypical urban population, raising concerns about the unchecked and unrestricted availability of paraquat.

### Clinical findings

As presented in the cases above, paraquat has many manifestations that range from mild early in the illness, to severe as the toxin spreads throughout the body. The determinants of the development of such manifestations will be discussed later. Focusing on the manifestations themselves, they can be divided into the following groups based on the systems involved – gastrointestinal, pulmonary, hepatobiliary, renal, and neurological. The various signs, symptoms, and important complications of the patients have been summarized in Table 1 given at the end of the case presentation section, and Table 2, given below.

**Table 1:** Summary of patient signs and symptoms

Case	Ulceration of the mouth or tongue	Abdominal pain	Vomiting	Dry cough	Odynophagia or dysphagia	Diarrhea	Throat pain	Other
1	N/A	N/A	Present	N/A	Present	N/A	Present	N/A
2	Present	N/A	Present	Present	Present	N/A	N/A	N/A
3	N/A	Present	Present	N/A	N/A	Present	Present	Left-sided chest pain, fever, chills, green-colored urine and stool
4	N/A	Present	Present	N/A	N/A	N/A	N/A	Dry oral cavity with hoarseness of voice
5	Present	Present	Present	Present	Present	N/A	Present	N/A
6	Present	N/A	Present	N/A	N/A	Present	N/A	N/A
7	Present	Present	Present	N/A	Present	N/A	N/A	Pain in oral cavity
8	N/A	N/A	Present	N/A	N/A	N/A	N/A	N/A
9	N/A	N/A	N/A	N/A	N/A	N/A	N/A	Burning sensation in throat
10	N/A	N/A	N/A	N/A	N/A	N/A	N/A	N/A
11	N/A	N/A	Present	N/A	N/A	N/A	N/A	N/A
12	N/A	N/A	Present	N/A	N/A	N/A	N/A	Glossitis, oral mucosa congestion
13	N/A	N/A	Present	N/A	N/A	N/A	N/A	Drowsy, gasping, cold extremities, left pupil dilated
14	N/A	N/A	N/A	N/A	N/A	N/A	N/A	Diffuse chest pain
15	Present	Present	N/A	N/A	N/A	N/A	Present	N/A

**Table 2:** Incidence of AKI, ALI, and pulmonary complications

Case	Acute kidney injury	Acute liver injury	Pulmonary complications
1	+	–	+
2	+	–	+
3	+	–	–
4	+	+	–
5	+	–	–
6	+	–	–
7	+	+	–
8	+	+	–
9	–	–	–
10	–	–	–
11	–	–	–
12	+	–	–
13	–	+	–
14	–	–	–
15	+	+	+

Starting with the most fatal, pulmonary concentrations of paraquat can reach higher than 10–20 times that of plasma concentrations due to greater prevalence of the polyamine uptake system in the alveoli^[8]^. These can manifest in the form of diffuse alveolitis, and pneumothorax in the first few days to pulmonary fibrosis, a hallmark of paraquat poisoning. Acute respiratory distress syndrome (ARDS) may set in as a complication about 24–48 hours of exposure to paraquat^[9]^. In fatal cases, histopathology has revealed pulmonary findings such as pulmonary congestion, mediastinal edema, and bleeding causing extensive pulmonary fibrosis^[10]^. Radiologically, these may appear as pleural thickening, subpleural reticular densities, pleural effusions, and sometimes segmented atelectasis as well. Together these manifestations may be called paraquat lung^[9]^. The choices of examinations usually include X-rays and CT scans. Cases 1, 2, and 3 discussed above show some of these complications, although none of the patients developed ARDS. This indicates that perhaps the poison was filtered out through dialysis before it could reach fatal concentrations and that the provided antioxidant therapy was effective. Other rare manifestations include pneumopericardium, pneumomediastinum (as mentioned earlier), pneumothorax, and subcutaneous emphysema^[11]^.

Paraquat, being a caustic compound, causes corrosive lesions of the mucosa along the gastrointestinal tract upon ingestion. These manifestations can range from mucosal lesions of the mouth and tongue, as mentioned before, called the “Paraquat tongue,” to esophageal ulcerations resulting in pain and dysphagia. These symptoms can occur not only due to the initial ingestion of the substance but also from reexposure, either when the patient tries to vomit it out – whether induced or as a result of the poison itself. Most of these lesions play little role in the determination of further prognosis as these occur even with minimal exposure to the poison and can be treated similarly to other caustic lesions. Some of the complications arising from esophageal ulceration may include perforation of the wall resulting in mediastinitis, bleeding, and pneumomediastinum, the latter being an indicator of very prognosis with a mortality rate of almost 100%^[12]^. Thirteen out of the 15 cases mentioned above were noted to have gastrointestinal manifestations, with ulceration of the mucosa, multiple episodes of spontaneous and induced vomiting, and some with bloody vomitus as well.

While there have been small amounts of paraquat discovered as a PM (post-mortem) finding in the bile of some patients indicating some role of the bile in its excretion, about 98% of paraquat is eliminated through the kidneys^[13]^. This means that good renal function is imperative in eliminating paraquat. Animal models have shown that as small as a 5% decline in renal function can result in an increase of plasma paraquat by five times^[14]^. Therefore, the heavy load of paraquat taken by the kidney can result in an AKI due to the large amounts of reactive oxygen species (ROS) released by the substance. This may manifest as reduced GFR, increased serum creatinine, and even oliguria. In cases with large doses, it may also result in acute tubular necrosis leading to acute renal failure^[15]^. Nine out of the 15 cases discussed above had developed an AKI due to paraquat poisoning. The incidence of AKIs in paraquat poisoning cases is about 50%^[16]^. The paraquat re-entering the system can also result in more pulmonary and hepatic complications. The liver is the primary organ for metabolizing paraquat found and increasing in peripheral blood. This is due to the liver containing the main sources of intrinsic antioxidants like Glutathione. Thus, the liver becomes a major target in paraquat poisoning, resulting in ALI^[17]^. This may manifest as jaundice, vomiting, metabolic disorders, and altered LFTs like elevated serum transaminases. Cases 4, 7, 8, 13, and 15, all developed an ALI with elevations of TB of 1.4 mg/dL and above. Therefore, the kidney and liver play major roles in the elimination, absorption, and metabolism of paraquat.

Neurotoxicity associated with paraquat is one of the most conclusive complications with multiple studies showing evidence of exposure to paraquat linking to Parkinson’s disease. However, this occurs on chronic exposure, not acute toxicity^[18]^. Within the brain, paraquat can be hypothesized to cause neuroinflammation in two ways – one is directly through ROS-induced damage and the other is due to induction of IL-6. Both instances have been shown to increase iron, resulting in long-term complications linked to various neurodegenerative disorders^[18,19]^. As of yet, the course of acute neurotoxicity caused by paraquat exposure is unclear, however, some patients shown to have the MRI changes consistent with that of acute neurotoxicity, presented with spasms and seizures^[20]^. None of our patients had any of these manifestations, they may nonetheless have been symptomless. Hence, none of the patients were investigated for acute paraquat neurotoxicity through MRI or other imaging studies. This was also partly due to the lack of facilities at the hospital and the lack of awareness around acute paraquat neurotoxicity.

Another serious yet rare manifestation is dilated cardiomyopathy (DCM)^[21]^, caused indirectly through increased vascular permeability, later complicating into heart failure. None of our patients presented with DCM or developed heart failure at the time of discharge, heart failure being a long-term complication.

### Prognosis

The prognosis of paraquat poisoning is generally considered poor and can be a combination of many different elements. Various factors have been proposed for predicting the prognosis. Some of these include – the amount ingested, duration of the development of complications, age, vomiting on early presentation, the association between fainting on admission and fatality, consumption to hospital time, etc^[22–24]^. Studying each factor individually is also a difficult endeavor considering they are all interdependent; the amount ingested, ingestion to hospital time, etc, have been summarized in Table 4.

An amount of higher than 40 mg/kg of paraquat ingested is considered fatal, causing development of MODS followed by death within a period of 2 days, while less than 20 mg/kg is considered to have generally good to moderate prognosis, with mild symptoms^[4]^. An amount between 20 and 40 mg/kg of paraquat is generally considered to have a very poor prognosis, with severe damage to the mucosa followed by MODS in many patients, while the survivors are also noted to have a poor prognosis^[4]^. They may develop pulmonary fibrosis, as described earlier, as a later complication within a period of 2–4 weeks, resulting in eventual death. Since all cases in this series involve self-harm and ER admissions, the exact ingestion amount is uncertain, relying solely on patient and guardian reports. Chi-square tests were performed to establish trends between dosage consumed and prognosis, as well as the ingestion-to-hospitalization and prognosis. It yielded a *P*-value of 0.935 and 0.665, respectively, indicating insignificant association between them (Table 3). Patients with >10 days of hospitalization were considered to be having bad prognosis as all the patients in this series are survivors. For the Chi-square test, patients were categorized as follows:

- Amount ingested: ≤20 mL, ≥20 mL, and unknown.
- Ingestion-to-hospitalization time: ≤ 6 hours and ≥ 6 hours.

**Table 3:** Comparison between amount ingested, ingestion to hospital time, and prognosis

Amount ingested	Good prognosis	Bad prognosis	*P*
≤20 mL	3	2	0.935
>20 mL	3	3	
Unknown	2	2	
Total	8	7	15
**Ingestion to hospitalization time**	**Good prognosis**	**Bad prognosis**	
≤6 hours	5	3	0.665
>6 hours	3	1	
Total	8	4	12

**Table 4:** Patient records: amount ingested, time to hospital admission, and duration of stay

Case	Amount ingested	Ingestion to hospitalization time	Duration of hospital stay
1	Unknown	6 hours	21 days
2	Unknown	Referred to this hospital 96 hours post-consumption	29 days
3	100 mL	19 hours	20 days
4	10 mL	6 hours	8 days
5	20 mL	75 hours	14 days
6	30 mL	3 hours	19 days
7	50 mL	24 hours	45 days
8	50 mL	3 hours	15 days
9	10 mL	4 hours	8 days
10	50 mL	3 hours	7 days
11	15 mL	4 hours	3 days
12	20 mL	Referred to this hospital 33 hours post-consumption	18 days
13	Unknown	12 hours	8 days
14	Unknown	4 hours	6 days
15	30 mL	Presented to local hospital within 4 hours	8 days
		Referred to this hospital 168 hours post-consumption	

Three patients (Cases 2, 12, and 15) were excluded from the ingestion-to-hospitalization time section, as they were not direct admissions to this hospital.

Similarly with reference to the amount ingested (Table 4), the symptoms and the observable clinical complications, some of our patients can be noted to have a positive correlation, excluding the cases with unknown amounts (Cases 1, 2, 13, 14). This is consistent with the findings of the study above by Dinis-Oliveira *et al*. As mentioned above, determination of the exact dose ingested by the patient is difficult as it completely depends on the patient’s/guardian’s testimonies. However, as seen in Cases 3, 4, 5, 6, 7, 8, 9, 11, 12, and 15, taking the ingestion to hospitalization time into consideration, there is a steady pattern with correspondence to the amount ingested, with increase in frequency and severity of both symptoms as well as the development of complications. Case 10 may be an example of a patient with an incorrect amount recorded/testified. While the patient is said to have consumed 50 mL of paraquat, the patient had no remarkable symptoms or complications. He also did not receive targeted antioxidant therapy or hemodialysis and was discharged within 7 days. While, Case 8, another patient with the same amount, 50 mL, and the same ingestion to hospitalization time, 3 hours, had significant symptoms along with both hepatic and renal complications. This patient received antioxidant therapy in the form of NAC and three rounds of hemodialysis, being discharged in a period of 15 days.

Delirrad *et al* (2015) reported that the shorter the time for the development of complications that is, the faster the admission to the ICU, the worse is the prognosis. Ten out of 15 of our cases developed pulmonary, renal and/or hepatic complications during their hospital stay at varying times, all have been noted to have poor prognosis by the healthcare provider in-charge at the time. This can perhaps be attributed to having a higher plasma paraquat concentration causing MODS by accumulating and causing higher damage through ROS. Studies have also discussed vomiting as a prognostic factor, however, in some studies, it has been shown to have a weak association^[25]^, and in some, a strong one^[26]^. Our study also shows a strong association, with 12 out of 15 cases having at least 1 episode of vomiting, 9 of which were not induced, the same cases that were noted earlier to have a poor prognosis by their attending physicians due to development of complications.

Age has also been brought up as a significant prognostic marker by Sabzghabaee *et al* (2010). Excluding the outliers (Cases 10 and 11), the mean age for our cases is 24.6 years, with a median of 26. Our study also reports similarly, Cases 1 (43 years) and 14 (38 years) as well as Case 7 (17 years) had poor prognosis. However, further in-depth analysis is needed to study the relation between the two. Sabzghabaee *et al* also reported on the paraquat ingestion to hospital time as a factor determining the prognosis, shown similarly in our study as well. Patients who had a longer ingestion to hospital time were noted to have poor prognosis, as seen for example with Case 7. Case 7, in particular, is a good example of all the prognostic factors working together in producing a poor prognosis, with an extreme of age, combined with a large amount of ingestion, having a longer ingestion to hospital time, vomiting as well as development of complications, However, it also underlines how difficult it is to understand their effect individually on the prognosis.

Further research could explore the impact of early treatment, as multiple studies have questioned the efficacy of symptomatic interventions namely gastric lavage, activated charcoal, hemodialysis, hemoperfusion, and cyclophosphamide in determining the prognosis^[25,27]^. However, they are considered the unofficial protocol in most hospitals in India, as done in our cases as well. Antioxidant therapy, including NAC, vitamins A and C, and steroids, may significantly impact prognosis, making early access crucial.

### Treatment

In cases of paraquat poisoning, a prompt and multifaceted management strategy is essential despite the absence of a definitive antidote. The initial therapeutic approach hinges on three critical goals: gastrointestinal decontamination, enhancing elimination, pulmonary protection^[28]^.

The therapeutic approach for paraquat intoxication primarily centers on supportive measures. Gastric lavage along with activated charcoal administration for gastrointestinal decontamination may be considered within the first 1–2 hours post-ingestion. This intervention aims to impede further systemic absorption of paraquat by binding to unabsorbed molecules within the gastrointestinal tract^[29]^. Upon arrival, nearly all patients in this case series underwent a standardized initial treatment approach. This involved gastric lavage using a nasogastric (NG) tube to remove any paraquat remaining in the stomach. A Foley catheter was also inserted to manage the urine output. Since these patients were unable to eat or drink anything by mouth (nil per oral), they received essential nutrients for recovery through a feeding tube (Ryle’s tube). Finally, they were administered intravenous fluids including normal saline and Ringer’s lactate solution to restore hydration and electrolyte balance.

The therapeutic approach to paraquat intoxication might be broadened to incorporate adjunctive immunosuppressive regimens, such as dexamethasone and cyclophosphamide. Additionally, the timely administration (within 2 hours of ingestion) of antioxidant therapy, encompassing N-acetylcysteine (NAC) and vitamins A, C, and E, alongside hemoperfusion, a blood purification technique, has demonstrated potential benefit in some studies^[2]^. The case series explored the use of various medications in treating paraquat poisoning. Among the 15 patients, 11 (73.34%) patients received NAC, a potential therapy to reduce cellular damage. Additionally, 7 (46.67%) patients were administered cyclophosphamide, while 10 (66.67%) received methylprednisolone, an anti-inflammatory medication. Notably, four (26.67%) patients received a combination of methylprednisolone and dexamethasone, another anti-inflammatory drug. One (6.67%) patient solely received dexamethasone. It’s important to note that nearly all patients were also given antioxidant supplements, including vitamins A, C, and E.

NAC is classified as a low-molecular-weight thiol. It demonstrates potent free radical-scavenging properties and participates in Redox reactions, acting as a potent antioxidant within the body. NAC stabilizes the free radicals and prevents them from causing cellular damage, effectively terminating their destructive potential.

Administration of corticosteroids can mitigate inflammatory processes associated with paraquat intoxication^[31]^. Consequently, steroid therapy might prove effective in treating paraquat poisoning after conventional treatments have failed, since lung inflammation could lead to a significant development of life-threatening hypoxemia observed in patients^[30]^. The use of NAC along with corticosteroids in our patients has been given in Table 5.

Hemodialysis maintains hemodynamic and metabolic stability in kidney failure through extracorporeal circulation, while hemoperfusion removes toxins and inflammatory mediators via adsorption^[31]^. These interventions effectively remove paraquat and its harmful metabolites from the circulation, minimizing their detrimental effects on various organs. A delay in initiating blood purification allows paraquat and its metabolites to accumulate within the body. This accumulation leads to progressively more severe tissue damage and complicates the subsequent removal of toxins, ultimately increasing the risk of mortality^[31]^. Among the 15 patients, dialysis treatment was administered to a total of 11 patients (73.3%) for varying durations. Four patients underwent the greatest number of cycles having received four cycles each, three received three cycles and the remaining four patients received two cycles. Notably, all patients who underwent dialysis experienced a normalization of their serum creatinine levels after treatment.

Emerging research shows that sodium salicylate (NaSAL) inhibits NF-κB, neutralizes ROS, reduces tissue injury by inhibiting MPO, prevents platelet aggregation, and protects lung cells from apoptosis. These diverse actions suggest NaSAL’s potential as a valuable therapeutic strategy for counteracting the severe effects of paraquat (PQ) intoxication. In a rat PQ intoxication model, high-dose NaSAL demonstrated remarkable efficacy, achieving 100% survival in treated rats compared to complete mortality in the control group by day 6. However, the lack of a commercially available parenteral NaSAL formulation necessitated exploring alternative options. This study aimed to evaluate the feasibility of lysine acetylsalicylate, a readily available prodrug of salicylate, as a potential antidote for PQ intoxication due to its suitability for parenteral administration^[32]^.

The variation in treatment modalities across different cases may have been influenced by several factors, such as the severity of poisoning at the time of presentation as given in the clinical features and treatment sections, the timing of medical intervention, the patient’s overall health status, and the availability of specific treatment options, operating in resource-limited settings. Differences in clinical judgment, and access to specialized care also contributed to the varied approaches. To establish more objective and consistent treatment criteria, it would be valuable to systematically analyze these factors across a larger cohort of cases, instead of the current data set of 15 patients. This could help identify key determinants in the selection of treatment strategies and form the basis for evidence-based, standardized guidelines that ensure more uniform care for patients with paraquat poisoning.

### The ban

Taking into consideration the burden of the disease due to self-consumption and the dangerous effects of this poison, more than 60 countries including; United Kingdom, China, Germany, South Korea, and Switzerland have banned this compound^[33]^. The implementation of this ban in Taiwan led to an estimated reduction of 58% of paraquat-associated suicides, leading to an overall reduction of pesticidal suicides by 37%^[34]^. Similar trends were also observed in South Korea and Sri Lanka, where there were reductions of 46% and 50% in pesticide-associated suicides respectively^[35,36]^. Also, the curbs on this deadly drug have proven to reduce case fatality rates of overall suicides in Malaysia from 21.2% to 17.3%^[37]^. However, this prohibition of the paraquat has not shown any negative effects on agricultural production in multiple studies that are based in South Korea, Vietnam, and India^[36]^.

### Limitations

The data used in this analysis may be incomplete as this is a retrospective study, where details might not be perfectly preserved or accurately documented. The lack of standardized treatment protocols in our study meant that patient management varied significantly depending on the clinical judgment and evaluation of the medical team involved. While the variations underscore the individualized nature of patient care, they also highlight the need for future studies to establish objective and standardized guidelines to minimize such inconsistencies and enable more robust comparisons of treatment outcomes.

Furthermore, this analysis was conducted in a resource-limited setting, which might limit its applicability to other regions with different healthcare infrastructures and resources. Finally, the lack of long-term patient follow-up restricted a comprehensive understanding of long-term health outcomes and the potential for chronic complications. This limitation underscores the need for more extensive, prospective studies and better data preservation methods to enhance our understanding of paraquat poisoning and improve patient care and outcomes on a broader scale. The amount ingested being analyzed in this study is subject to recall bias as it depended solely on the patient or the guardian’s testimonials.

## Conclusion

Paraquat poisoning poses a substantial health risk, and while all cases in this analysis survived, the lack of a standardized management protocol restricts our understanding of the factors that are at play here. Further research is crucial to establish optimal treatment strategies and to explore the potential for long-term complications which are not well understood. The study provides a brief understanding of paraquat poisoning, highlighting current gaps in knowledge and emphasizing the need for ongoing research. The 15 survivors who were studied here share a few common features that might have influenced their good prognosis like, early initiation of Immunosuppressive medications, performing dialysis at a lower threshold, and also lesser ingestion to hospitalization time. However, these cannot be standardized without more comprehensive clinical trials. Continuing research efforts and implementing stricter safety measures for paraquat use, including potential restrictions on its availability due to its well-documented link to self-harm and suicide are essential steps toward reducing poisoning incidents and enhancing long-term outcomes for affected patients. These measures promise to foster a better understanding of paraquat toxicity and improve overall patient care and prognosis.

## Data Availability

The dataset regarding the case series can be submitted to the journal on request.
